# Structural Changes Induced in Grapevine (*Vitis vinifera* L.) DNA by Femtosecond IR Laser Pulses: A Surface-Enhanced Raman Spectroscopic Study

**DOI:** 10.3390/nano6060096

**Published:** 2016-05-25

**Authors:** Nicoleta E. Dina, Cristina M. Muntean, Nicolae Leopold, Alexandra Fălămaș, Adela Halmagyi, Ana Coste

**Affiliations:** 1 National Institute for Research & Development of Isotopic and Molecular Technologies, Donat 67-103, 400293 Cluj-Napoca, Romania; nicoleta.dina@itim-cj.ro (N.E.D.); alexandra.falamas@itim-cj.ro (A.F.); 2 Babeş-Bolyai University, Faculty of Physics, Kogălniceanu 1, 400084 Cluj-Napoca, Romania; nicolae.leopold@phys.ubbcluj.ro; 3 National Institute of Research and Development for Biological Sciences, branch Institute of Biological Research, Republicii Street 48, 400015 Cluj-Napoca, Romania; adela.halmagyi@icbcluj.ro (A.H.); ana.coste@icbcluj.ro (A.C.)

**Keywords:** surface-enhanced Raman spectroscopy, genomic DNA, silver colloid, femtosecond infrared (IR) laser pulses, principal component analysis discrimination

## Abstract

In this work, surface-enhanced Raman spectra of ten genomic DNAs extracted from leaf tissues of different grapevine (*Vitis vinifera* L.) varieties, respectively, are analyzed in the wavenumber range 300–1800 cm^−1^. Furthermore, structural changes induced in grapevine genomic nucleic acids upon femtosecond (170 fs) infrared (IR) laser pulse irradiation (λ = 1100 nm) are discussed in detail for seven genomic DNAs, respectively. Surface-enhanced Raman spectroscopy (SERS) signatures, vibrational band assignments and structural characterization of genomic DNAs are reported for each case. As a general observation, the wavenumber range between 1500 and 1660 cm^−1^ of the spectra seems to be modified upon laser treatment. This finding could reflect changes in the base-stacking interactions in DNA. Spectral shifts are mainly attributed to purines (dA, dG) and deoxyribose. Pyrimidine residues seem to be less affected by IR femtosecond laser pulse irradiation. Furthermore, changes in the conformational properties of nucleic acid segments are observed after laser treatment. We have found that DNA isolated from Feteasca Neagra grapevine leaf tissues is the most structurally-responsive system to the femtosecond IR laser irradiation process. In addition, using unbiased computational resources by means of principal component analysis (PCA), eight different grapevine varieties were discriminated.

## 1. Introduction

Nowadays, it is established that DNA is one of the principal macromolecular targets for radiation damage. Any agent that causes DNA damage has potential applications in cancer therapy, since cancer is a disease of DNA, caused mainly by alterations of the genome. Femtosecond laser technology offers potential new insights that could be used in this research.

Particularly, molecular mechanisms of the biological action of femtosecond IR pulsed irradiation on DNA have not yet been completely established. Previously, it was found that DNA strand breaks are induced via nonlinear excitation with femtosecond laser pulses at λ = 1050 nm [1]. The selective photo-disruption of the nucleus of a single living cell, without modifying the cell morphology, is also demonstrated in living human breast cells using near-infrared (NIR) (790 nm) femtosecond (110 fs) Ti:sapphire laser pulses [2]. Besides, some results suggest that two-wavelength femtosecond laser pulses are well suited for the identification of transcription factors interacting with defined sequences and for studying the kinetics of protein-nucleic acid interactions in intact cells [3].

The development of simple, reliable, high-throughput methods to detect structural variations in DNA is crucial for the development of DNA-based diagnosis and forensics [4]. Particularly, surface-enhanced Raman spectroscopy (SERS) takes advantage of the strongly increased Raman signals generated by local field enhancement near metallic (typically Au and Ag) nanostructures. The general mechanisms of SERS, together with the geometry requirements of the metallic environment for generating maximum enhancement, such as “hot spots”, have been studied in some detail [5]. As compared to normal Raman signals, SERS scattering cross-sections of molecules residing at or near the surface of roughened or nanostructured materials may be enhanced by factors up to ~10^14^ [6]. The SERS phenomenon is also capable of detecting various structural, thermodynamic and kinetic properties of DNA and is not limited by molecular size or state of aggregation. Apart from the molecular information, insight into the various electrostatic, hydrophobic and hydrogen bonding interactions between the nucleotides is given [7,8].

In this work, surface-enhanced Raman spectral characteristics of genomic DNAs isolated from leaves of different grapevine (*Vitis vinifera* L.) varieties (Feteasca Neagra, Tamaioasa Romaneasca, Gordan, Feteasca Regala, Coarna Neagra, Ardeleanca, Braghina, Carloganca, Francuse and Cramposie) have been studied between 300 and 1800 cm^−1^. Furthermore, structural changes induced in genomic DNAs upon femtosecond (170 fs) IR irradiation (λ = 1100 nm) are discussed in detail for seven genomic DNAs extracted from Feteasca Regala, Braghina, Francuse, Carloganca, Feteasca Neagra, Tamaioasa Romaneasca and Ardeleanca grapevine varieties, respectively. Ultraviolet-visible spectrophotometry (UV-Vis) characterization of DNA-silver nanoparticles systems is presented in the Supplementary Materials (Figure S1 and Figure S2).

In addition, using unbiased computational resources by means of principal component analysis (PCA), eight grapevine varieties were discriminated. Qiu *et al.* have recently (2014) reported a joint study on plant genomic DNAs by using hierarchical cluster analysis (HCA) and principal component analysis (PCA) on SERS spectra recorded from nine species of plants [9]. However, as far as we know, a study including SERS spectra acquisition on DNA extracted from grapevine varieties and Raman monitoring of the DNA structural response to femtosecond IR pulse irradiation was not yet reported.

Moreover, part of this study could be used as a prerequisite for future research investigations on tumor DNA, in the same irradiation conditions, since at the chemical level, all DNA molecules are made from the same four chemical building blocks, called nucleotides. Cancer research could benefit from ultra-fast laser technology by investigating the impact of femtosecond laser pulses on tumoral DNA, on normal DNA and on cancerous cells, respectively, minimizing the risk of DNA anomalies being induced.

## 2. Materials and Methods

### 2.1. DNA Extraction

Ten grapevine (*Vitis vinifera* L.) varieties (Feteasca Neagra, Tamaioasa Romaneasca, Gordan, Feteasca Regala, Coarna Neagra, Ardeleanca, Braghina, Carloganca, Francuse and Cramposie) provided by the Research and Development Stations for Viticulture and Enology from Dragasani, Romania, were sampled. Total genomic DNA was extracted from 20 mg of silica gel dried leaf material, using the DNeasy Plant Mini Kit (QIAGEN Inc., Valencia, CA, USA) and protocols of Qiagen. The purified total DNA was quantified using the NanoDrop 2000 Spectrophotometer (Thermo Scientific, Wilmington, DE, USA). DNA samples were stored at −80 °C until examination. For SERS measurements, genomic nucleic acids were suspended in ultrapure water. Non-irradiated and femtosecond IR laser pulses-treated DNAs were analyzed, respectively.

### 2.2. Surface-Enhanced Raman Spectroscopy

#### 2.2.1. Preparation of DNA-Nanoparticle Complexes

Metallic nanoparticles with an estimated medium diameter of 25 nm have been obtained [10,11]. We have described in detail elsewhere the method used for the preparation of Ag colloidal SERS substrate, by reducing Ag^+^ with hydroxylamine [8,12]. Samples were prepared by adding aliquots of stock DNA solutions, respectively, to 500 μL silver colloid, respectively, into a 1-mL glass cuvette. The final DNA amount in each colloidal suspension for SERS measurements is shown in Tables S1 and S2 (Supplementary Materials). The pH value of DNA-nanoparticle complexes was 7. Any change of color was observed in the nucleic acids-Ag substrate systems prepared for this surface-enhanced Raman spectroscopic study [10,12].

#### 2.2.2. Instrumentation

A Delta Nu Advantage spectrometer (Delta Nu, Laramie, WY, USA), equipped with a doubled frequency Nd:YAG laser emitting at 532 nm and 4.5 mW laser power, was used for SERS measurements. Spectra were obtained by averaging 5 recordings using the dedicated software NuSpec (Delta Nu, Laramie, WY, USA). The silver colloid was Raman tested. No Raman active bands were observed in the recorded spectrum. The reference suspension obtained by adding 10 μL of ultrapure water in 500 μL silver colloid into a 1-mL glass cuvette was also Raman tested. The reference (ultrapure water in colloid) spectrum was subtracted from the surface-enhanced Raman spectra of the Ag-DNA systems, respectively, and baseline correction was applied.

### 2.3. Femtosecond Laser System

A Yb:KGW femtosecond laser system (Light Conversion, Vilnius, Lithuania) (Pharos) emitting pulses of a 170-fs duration at 1030 nm was used. The repetition frequency of the laser was 80 kHz, and 75 μJ energy per pulse was used. The 1030-nm pulse is used to pump a collinear optical parametric amplifier (Light Conversion, Vilnius, Lithuania) (Orpheus), which yields tunable output between 620 and 2600 nm. Additional frequency mixing units can generate second and fourth harmonics of signal and idler beams, extending the total emission range down to 210 nm.

DNA molecules suspended in ultrapure water were irradiated, respectively, with λ = 1100 nm, 60 mW average power, for 30 min. The radiant fluence was 6.8 × 10^−6^ J/cm^2^. The irradiation set-up consisted of mirrors for guiding the laser beam to the irradiation spot, which is adapted for holding Eppendorf tubes. The laser spot irradiated the central area of the tube.

### 2.4. UV-Vis Characterization of DNA-Silver Nanoparticle Complexes

Ultraviolet-visible spectrophotometry (UV-Vis) characterization of DNA-silver nanoparticles systems is presented in the Supplementary Materials (Figure S1 and Figure S2).

### 2.5. Computational Details

The software Unscrambler^®^ X Version 10.1 (Camo Software AS., Oslo, Norway) was used to perform the principal component analysis (PCA). The pre-processing of the DNA samples database consisted of using multiplicative scattering correction (MSC) prior to the PCA analysis.

## 3. Results and Discussion

### 3.1. Surface-Enhanced Raman Spectroscopy of DNA Extracted from Grapevine Leaf Tissues

**Figure 1:**
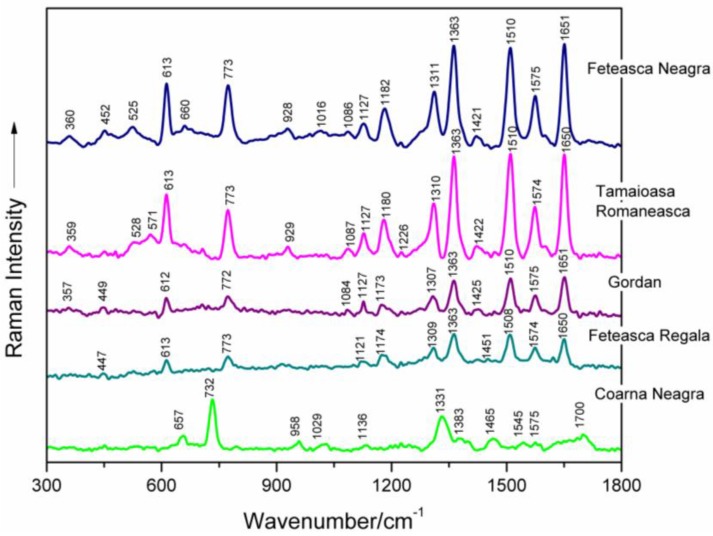
Surface-enhanced Raman spectroscopy (SERS) spectra of five selected genomic DNAs isolated from leaves of different grapevine varieties (Feteasca Neagra, Tamaioasa Romaneasca, Gordan, Feteasca Regala, Coarna Neagra), respectively, as labeled in the figure.

**Figure 2:**
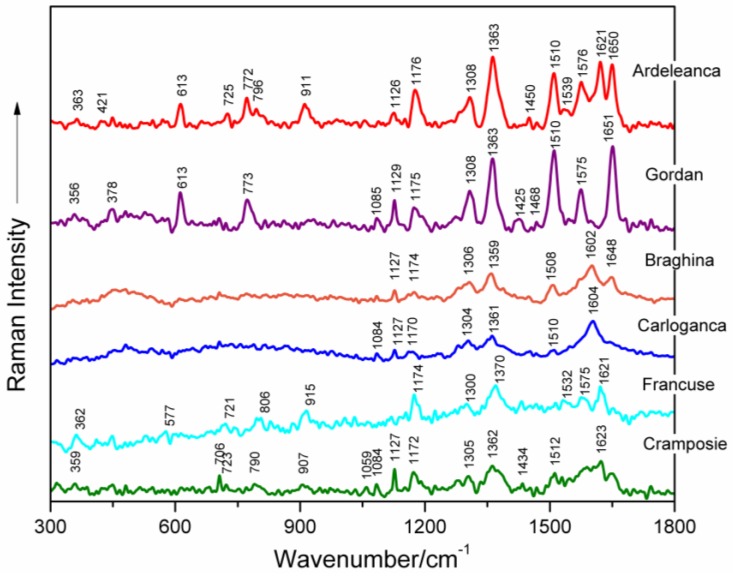
SERS spectra of six selected genomic DNAs isolated from leaves of different grapevine varieties (Ardeleanca, Gordan, Braghina, Carloganca, Francuse, Cramposie), respectively, as labeled in the figure.

Surface-enhanced Raman signatures of five selected DNAs extracted from different grapevine varieties (Feteasca Neagra, Tamaioasa Romaneasca, Gordan, Feteasca Regala and Coarna Neagra) are illustrated in Figure 1 between 300 and 1800 cm^−1^. The most important marker bands in the spectra are labelled. Besides, SERS profiles of six selected grapevine DNAs (from Ardeleanca, Gordan, Braghina, Carloganca, Francuse and Cramposie, respectively) are depicted in Figure 2, in the wavenumber range 300–1800 cm^−1^.

Table 1 presents the wavenumbers and corresponding assignments of the surface-enhanced Raman bands, which characterize each spectrum of the investigated genomic DNAs from different grapevine varieties (Figure 1 and Figure 2) [8,9,10,12,13,14,15,16,17,18,19,20,21,22,23].

In the following, selective surface-enhanced Raman modes will be analyzed.

Concerted ring vibrations of base residues, often associated with modes of the glycosidic bond and possibly with modes of bonds within the deoxyribose ring, are to be found between 600 and 800 cm^−1^ [10,16,24]. Particularly, the narrow SERS band appearing near 613 cm^−1^ in all spectra of grapevine varieties DNAs, being very stable in wavenumber, is due to a vibration localized in the deoxyribose (C3′-*endo-*anti) [25]. A weak marker band is noticed around 706 cm^−1^, only in the case of Tamaioasa Romaneasca, Braghina and Cramposie grapevine DNA spectra. It might be assigned to dA ring breathing (Figure 1 and Figure 2, Table 1) [9,10,21,24,26,27,28,29]. The SERS spectral feature found near 773 cm^−1^ in six spectra of DNAs extracted from leaves of different grapevine varieties is originating from C2′-*endo*-anti nucleoside conformers of dC.

The marker band of deoxyribose C2′-*endo* conformation is centered on 829 cm^−1^ in the spectrum of Francuse DNA, being absent in other SERS data of grapevine varieties DNAs. The weak band detected at 913 cm^−1^ in the spectra of DNA from different grapevine leaf tissues and near 929 cm^−1^ in the spectra of genomic DNAs from Feteasca Neagra and Tamaioasa Romaneasca varieties, respectively, might be due to deoxyribose [18,21,22,25].

A low intensity SERS marker band is detected at 1086 cm^−1^ in the spectra of Feteasca Neagra, Tamaioasa Romaneasca, Gordan, Ardeleanca, Braghina, Carloganca and Cramposie DNAs, respectively. This spectral contribution is due to symmetric stretching vibration of the PO_2_^−^ moiety, being independent of the base sequence and composition [9,10,15,17,18,19,20,30,31,32,33,34,35,36,37,38]. The weak and very weak intensity of this band in the SERS spectra of grapevine DNAs can be a consequence of the adsorption properties of DNA to the silver colloid [10]. As a general observation, the phosphodioxy DNA marker band has a higher intensity in the normal Raman spectrum of nucleic acid [36].

In-plane overlapped Raman vibrations of DNA base residues are mainly seen between 1100 and 1600 cm^−1^ ([10,14,16] and the references therein). The band appearing in the wavenumber range 1119–1136 cm^−1^ for the SERS spectra of different grapevine varieties DNAs might be attributed to dA residues (C8–N9 stretching, N9–H, C8–H deformation) ([14,21,39] and the references therein). A Raman vibrational mode, with a major contribution from dA and dG residues (C5–C6 stretching) is found in the wavenumber range 1170–1182 cm^−1^ in the case of DNAs extracted from *Vitis Vinifera* L. leaf tissues. Further, the moderate or strong SERS bands appearing between 1300 and 1311 cm^−1^ for all of the investigated genomic DNAs, can be attributed to ring vibrations of adenine and guanine (see Figure 1 and Figure 2, Table 1) [15,17,18,19,21,40]. Besides, we suppose that the high intensity SERS vibration near 1363 cm^−1^, characterized by a very stable wavenumber for several analyzed DNA spectra, is due to ring stretching modes in dT, dA and dG residues [9,19,20,21,41,42]. Furthermore, a weak marker band is found at 1423 cm^−1^ in different spectra of DNA isolated from grapevine varieties, indicating the contribution of deoxyribose C2′H_2_ scissoring. A strong and wavenumber-stable SERS marker band, probably due to contributions from adenine residues (NH_2_ deformation), is found near 1510 cm^−1^ for several spectra illustrated in Figure 1 and Figure 2 [10,15,17,18,19]. The marker band found between 1574 and 1576 cm^−1^ (Table 1) is assigned to ring stretching vibrational modes in dG and dA residues [9,15,17,18,19,20].

In the next spectral range 1600–1750 cm^−1^, carbonyl stretching vibrations of dT and dG residues are expected to be found. Particularly, the intense SERS spectral feature, appearing between 1648 and 1651 cm^−1^ in the spectra of DNAs from grapevine leaf tissues, respectively, is mostly due to dT(C=O) and δ(H_2_O) vibrations ([9,10,17,18,20] and the references therein).

As a general observation, the SERS spectra of DNA molecules isolated from leaf tissues of different grapevine varieties, respectively, show precise profiles, providing useful information concerning molecular structure.

### 3.2. Effects of Femtosecond IR Laser Pulses on Genomic DNA Structure

**Figure 3:**
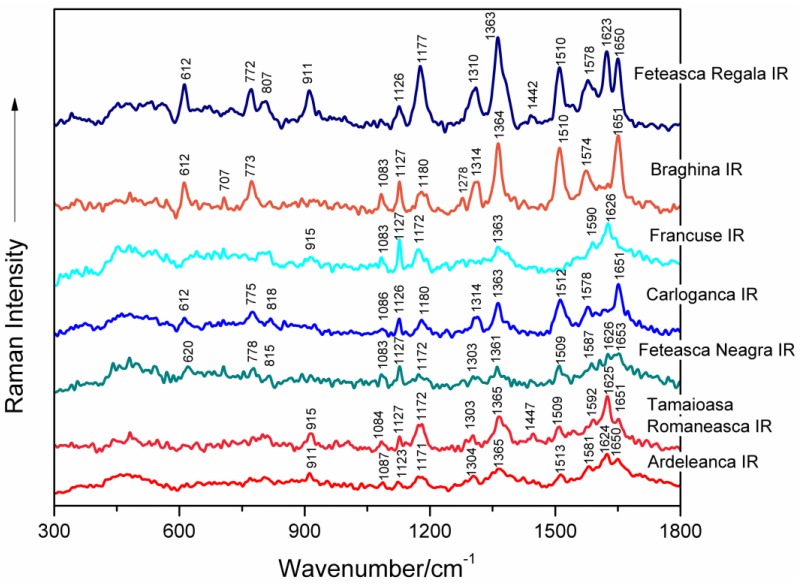
SERS spectra of seven genomic DNAs isolated from leaves of different grapevine varieties (Feteasca Regala, Braghina, Francuse, Carloganca, Feteasca Neagra, Tamaioasa Romaneasca, Ardeleanca), respectively, as labeled in the figure. DNA samples were irradiated with femtosecond infrared (IR) laser pulses (1100 nm, 60 mW average power, 30 min).

Surface-enhanced Raman spectral characteristics of seven genomic DNAs isolated from leaves of different grapevine varieties (Feteasca Regala, Braghina, Francuse, Carloganca, Feteasca Neagra, Tamaioasa Romaneasca, Ardeleanca) that were exhibiting a SERS signal after IR irradiation (1100 nm) are presented in Figure 3 between 300 and 1800 cm^−1^.

**Figure 4:**
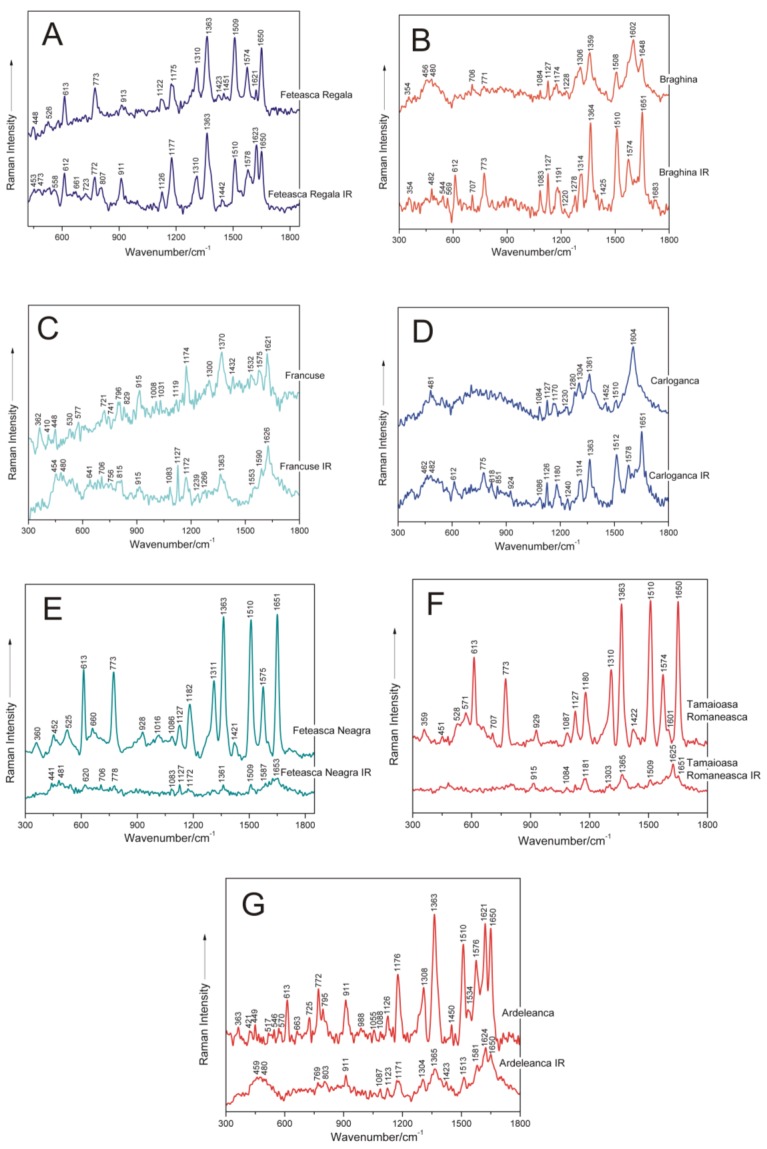
SERS spectra of seven non-irradiated and femtosecond IR laser pulse-treated genomic DNAs extracted from different grapevine varieties, respectively: **A**, Feteasca Regala; **B**, Braghina; **C**, Francuse; **D**, Carloganca; **E**, Feteasca Neagra; **F**, Tamaioasa Romaneasca; **G**, Ardeleanca.

Figure 4 shows, by comparison, the SERS spectra recorded from the DNA samples of seven grapevine varieties before and after IR irradiation with 1100 nm femtosecond (170 fs) laser pulses. A concise presentation of the SERS band wavenumbers and their tentative assignments, corresponding to IR-irradiated grapevine genomic DNAs, are shown in Table 2 [8,9,10,12,13,14,15,16,17,18,19,20,21,22].

Further in this work, changes induced by femtosecond IR laser pulses irradiation in the molecular structural level of genomic DNAs isolated from different grapevine varieties, respectively, as compared to the corresponding nucleic acids in the non-irradiated samples, are discussed (Figure 4).

Particularly, in the case of DNA isolated from the Feteasca Regala grapevine variety, the following SERS bands can be found, corresponding to both control and IR laser-treated samples (Figure 4A), respectively: 448 cm^−1^, 613 cm^−1^, 773 cm^−1^, 913 cm^−1^, 1122 cm^−1^, 1175 cm^−1^, 1310 cm^−1^, 1363 cm^−1^, 1423 cm^−1^, 1509 cm^−1^, 1574 cm^−1^, 1621 cm^−1^ and 1650 cm^−1^. The weak marker band found near 1451 cm^−1^ in the control DNA case, is missing from the spectrum of irradiated genomic DNA from the Feteasca Regala variety. Furthermore, a new band appears after femtosecond IR laser treatment of the biopolymer, centered at 807 cm^−1^, indicating a contribution of backbone 5′C–O–P–O–C3′ symmetric stretching. Furthermore, the relative intensities I_1175_/I_1310_ and I_1621_/I_1650_ are modified in the spectrum of irradiated DNA (Figure 4A) as compared to the reference one. Besides, the band profile of the Feteasca Regala DNA spectrum, in the wavenumber range 1500–1655 cm^−1^, being mostly due to dG, dA, dC and dT residues, seems to suffer some changes upon the irradiation process. This finding can reflect changes in the base-stacking interactions in DNA. We have also observed at 1363 cm^−1^ a very stable band in wavenumber, attributed to dT, dA, dG (C–N stretching (pyrimidine)), indicating a low influence of femtosecond IR laser pulses at this structural level. For the Feteasca Regala grapevine variety DNA, the Raman shifts of the SERS bands are insignificant as compared to the corresponding spectrum of irradiated DNA. The only bands affected by laser treatment seem to be at 1423 cm^−1^ (deoxyribose C2′H_2_ scissoring) and near 1451 cm^−1^, since they are replaced by a single band at 1442 cm^−1^ (see Table 3).

For the Braghina grapevine variety DNA (Figure 4B), the reverse tendency is to be observed: the SERS bands of the irradiated DNA sample are more intense, than those for the control one. Moreover, there are additional spectral contributions only present in the SERS spectrum of irradiated DNA, as compared to the spectrum of control nucleic acid: 544 cm^−1^ (dC/dT (ring deformation)), 569 cm^−1^ (dA (C2–H, N9–H wagging)), 612 cm^−1^ (deoxyribose (C3′-*endo*-anti)), 1278 cm^−1^ (dC (C–NH_2_ stretching and ring stretching)), 1425 cm^−1^ (deoxyribose C2′H_2_ scissoring) and 1574 cm^−1^ (dG, dA (C2=N3 of guanine)). Furthermore, some changes in the conformational properties of these nucleic acid following femtosecond laser treatment are associated with the relative intensity of the bands near 612 cm^−1^ (deoxyribose, C3′-*endo*-anti) and 773 cm^−1^ (dC, C2′-*endo*-anti), respectively. Changes in the intensities ratio I_1314_/I_1364_ in the spectrum of irradiated DNA, as compared to the control one, are proof of modified C–N stretching in dA, dG and dT residues. The region between 1500 and 1683 cm^−1^ seems also to be affected in this case by the IR laser irradiation process. As an overall observation, Raman shifts of some marker modes are also detected in the SERS spectra of treated DNA, as compared to the non-irradiated ones, respectively (Table 3), e.g.: the band at 1174 cm^−1^ shifts to 1191 cm^−1^; 1228 cm^−1^ shifts near 1220 cm^−1^; 1306 cm^−1^ shifts around 1314 cm^−1^; and the band at 1602 cm^−1^ shifts to 1574 cm^−1^. These shifts are also proof of IR laser-induced structural changes in DNA extracted from Braghina grapevine variety leaves.

In the following, the effect of sample femtosecond IR laser pulse exposure is discussed for genomic DNA isolated from leaf tissues of the Francuse grapevine variety. The spectral feature near 915 cm^−1^ (deoxyribose) is not so notable in the SERS vibrational signature of DNA after laser treatment (Figure 4C). Furthermore, the relative intensity I_1119_/I_1174_ is modified in the spectrum of treated DNA (Figure 4), as compared to the reference one, being proof of changes in purinic dA and dG residues. Besides, the band at 1300 cm^−1^ attributed to dA and dG (C–N stretching (imidazole)) disappeared after the irradiation process. The SERS marker band, which corresponds to deoxyribose C2′H_2_ scissoring (1432 cm^−1^), respectively, was found to be not important for irradiated DNA from the Francuse variety. Furthermore, the spectral profile between 1500 and 1660 cm^−1^, including relative intensity I_1532_/I_1575_, seems to be modified after DNA treatment. The following SERS bands are shifted in the spectrum of irradiated DNA from Francuse, as compared to the control one: 721 cm^−1^ shifts to 706 cm^−1^; 741 cm^−1^ appears at 756 cm^−1^; 796 cm^−1^ shifts around 815 cm^−1^; 1119 cm^−1^ shifts to 1127 cm^−1^; 1532 cm^−1^ shifts near 1553 cm^−1^; and 1575 cm^−1^ shifts around 1590 cm^−1^, respectively. Other bands absent in the SERS spectrum of the DNA sample after irradiation are: 829 cm^−1^ (deoxyribose C2′-*endo*), 1008 cm^−1^ (dT (out-of-plane NH_2_ wagging)) and 1031 cm^−1^ (dA (N-deoxyribose stretching)).

For the Carloganca grapevine variety DNA, the intensity of the SERS marker bands is comparable for the two compared spectra (Figure 4D). The bands in the region 600–1050 cm^−1^ are better revealed in the spectrum of irradiated DNA, as compared to the reference one. These bands are present at 612 cm^−1^ (deoxyribose C3′-*endo-*anti), 775 cm^−1^ (dC, C2′-*endo*-anti), 818 cm^−1^ (backbone 5′C–O–P–O–C3′ symmetric stretching), 851 cm^−1^ (deoxyribose C3′-*endo*) and 924 cm^−1^ (deoxyribose), respectively. In the SERS spectrum of control DNA from Carloganca, two bands are observed at 1280 cm^−1^ (dC (C–NH_2_ stretching and ring stretching)) and near 1452 cm^−1^ (deoxyribose CH_2_), which are not found in the SERS spectrum of irradiated DNA. Besides, the relative intensities I_1084_/I_1127_ and I_1510_/I_1604_ seem to be modified in the spectrum of Carloganca IR (Figure 4D). Furthermore, significant Raman shifts of the SERS marker modes, characterizing non-irradiated DNA and laser-treated nucleic acid, respectively, are observed. The band near 1170 cm^−1^ is shifted to 1180 cm^−1^; the vibration at 1230 cm^−1^ is found around 1240 cm^−1^; the marker from 1304 cm^−1^ appears at 1314 cm^−1^; and the band near 1604 cm^−1^ is found around 1578 cm^−1^, respectively (Table 3). Furthermore, the vibrational profile in the wavenumber range 1500–1660 cm^−1^ seems to suffer several changes after the irradiation process, as compared to the control DNA spectrum.

The Feteasca Neagra grapevine variety DNA is the most structurally-responsive system to the IR laser irradiation process (Figure 4E). A lack of moderate and high intensity surface-enhanced Raman bands is to be observed in the vibrational profile of this treated DNA. Weak and very weak bands are only to be found for molecular subgroups in the proximity of silver nanoparticles. Furthermore, the SERS spectrum of control DNA exhibits several bands, which are missing in the SERS spectrum of irradiated DNA: 525 cm^−1^ (dC/dT (ring deformation)), 660 cm^−1^ (dG (ring breathing)), 928 cm^−1^ (deoxyribose), 1016 cm^−1^ (dT (out-of-plane NH_2_ wagging)), 1311 cm^−1^ (dA, dG (C–N stretching imidazole)) and 1421 cm^−1^ (deoxyribose C2′H_2_ scissoring), respectively. The Raman shifts observed by comparing the SERS spectrum of non-treated DNA to the SERS signature of IR-irradiated nucleic acid have been detected for the following bands: the marker near 452 cm^−1^ shifts to 441 cm^−1^; the band at 1182 cm^−1^ has been found around 1172 cm^−1^; and the mode near 1575 cm^−1^ was observed at 1587 cm^−1^, respectively. Loss of essential structural organization, including the alteration of the DNA double helix, might be found in this case.

DNA from Tamaioasa Romaneasca grapevine leaf tissue is another responsive system to IR femtosecond laser pulse treatment (Figure 4F). As a general observation, relative SERS band intensities of this DNA sample are highly modified after irradiation. The main SERS bands disappear, and two of them are shifted from 929 cm^−1^ (deoxyribose) to 915 cm^−1^ and from 1601 cm^−1^ (dA, dG, dC (NH_2_ deformation)) near 1625 cm^−1^, respectively (Table 3).

For the Ardeleanca grapevine variety, the bands present at 613 cm^−1^, 725 cm^−1^ and 1534 cm^−1^, respectively, in the spectrum of reference DNA disappear in the spectrum of the irradiated sample (Figure 4G). Besides, the relative intensity I_1176_/I_1308_ seems to be modified in the spectrum of Ardeleanca IR (Figure 4G). Both of these bands are attributed to purinic dA and dG residues. Particularly, the following spectral contributions present significant shifts upon IR femtosecond laser treatment, as compared to the control case: the mode at 449 cm^−1^ (stretching deoxyribose) is shifted to 459 cm^−1^; the band near 795 cm^−1^ (backbone 5′C–O–P–O–C3′ symmetric stretching) is shifted to 803 cm^−1^; and the marker near 1450 cm^−1^ (deoxyribose CH_2_) is shifted around 1423 cm^−1^. Previously, it was found that the bands detected between 1444 and 1464 cm^−1^ in the SERS spectra of plant DNAs are attributed to the CH_2_ scissor of deoxyribose [8,10,14,17,23,31,37].

A synthetic presentation of the SERS band shifts after DNA irradiation is presented in Table 3. As illustrated in this table and in the present subsection, spectral shifts are mainly attributed to purines (dA, dG) and deoxyribose. Pyrimidines residues seem to be less affected by irradiation with femtosecond IR laser pulses. Furthermore, changes in the conformational properties of nucleic acids are to be observed after laser treatment.

The laser-biological system interactions can induce several mechanisms, such as photochemical interactions, thermal interactions, photoablation or photodisruption. High intensity short pulsed laser irradiation has been recently used as a very attractive technique for tumor irradiation, as it produces highly localized heating at desired locations [43]. The heat generated in biological systems by high intensity lasers is dependent of the irradiation laser parameters, such as: the energy density, peak power, repetition rate and pulse length. Besides, the amount of heat generated is dependent on the optical properties of the system, like absorption and scattering coefficients.

Particularly, for aqueous systems, the absorption of water molecules is highly important in thermal effects, the absorption coefficient being dependent on the laser irradiation wavelength. In this study, we have chosen the 1100-nm wavelength, because water absorption is generally low in this spectral region, and moreover, most animal cells and tissues are considered transparent in the 700–1100 nm spectral region. The temperature increase due to the heating of water at the focus spot was found to be 1.4 °C–1.9 °C/100 mW for 1064-nm lasers [44].

DNA NIR irradiation studies have shown that the 1400-nm laser wavelength focused on a spot of 3 µm in diameter can induce DNA denaturation, due to the temperature rise created at the focal point. Hung *et al.* observed the denaturation for 30 mW laser power, which induced a temperature of 110 °C within the laser spot diameter [45]. However, this wavelength is further in the spectral region from the one used in our experiment. Besides, Jaunich *et al.* observed a rise in the temperature at the focal spot in skin phantoms, when irradiated using 1064 nm of 1.3 W, at a 1-kHz pulse repetition frequency, for 10 s [43]. They considered a value of 15 s for the thermal relaxation time and concluded that this is one of the governing parameters for bio-heat transfer analysis.

All of these studies indicate that the NIR irradiation used in our experiments might induce structural alterations of the DNA molecules and that one of the possible mechanisms is the temperature increase at the focal spot during irradiation. There are ongoing studies in our research group to elucidate these mechanisms.

In principle, spectral changes might be associated not only with irradiation-induced structural modifications in the case of DNA from grapevine varieties, but also with effects in the adsorption mechanisms of these nucleic acids on a silver surface [8]. Recently, Miljanić *et al.* (2015) studied by SERS the adsorption mechanisms of some nucleic acid components on Ag surfaces [46]. In this work, binding of adenosine 5′-monophosphate through the N1 atom and the NH_2_ group with Ag was found. Furthermore, adsorption of guanosine 5′-monophosphate on a silver surface via N1–H and C=O group was suggested. Besides, cytidine 5′-monophosphate favored adsorption on Ag colloid through the carbonyl oxygen [46]. Particularly, Miljanić *et al.* (2015) have mentioned that significant changes in relative band intensities of adenosine 5′-monophosphate with a concentration decrease were not observed, indicating only a minor change in the position of these molecules during the concentration-induced complex formation. Besides, the concentration change did not affect the adsorption mechanism of guanosine 5′-monophosphate molecules onto the Ag surface [46].

### 3.3. Principal Component Analysis on the SERS Spectra Recorded from Non-Irradiated Grapevine DNAs

The essential information from complex spectroscopic patterns is usually extracted by using chemometrics, such as principal component analysis (PCA) [47,48]. In order to assess the identification potential of DNAs extracted from different grapevine varieties, PCA was performed on the pre-processed SERS spectra collected from eight sets of samples (Ardeleanca, Gordan, Francuse, Cramposie, Feteasca Neagra, Feteasca Regala, Tamaioasa Romaneasca and Coarna Neagra). Firstly, the SERS spectra were quality tested, in order to assure that only the spectra with an appropriate signal to noise ratio are to be considered in the PCA analysis. Consequently, spectra for which the maximum of the most intense SERS band in the vibrational profile was less than 500 counts, respectively, were rejected. Therefore, only eight from ten samples were selected for PCA analysis. The final database was formed based on a set containing between 7 and 13 SERS spectra for each extracted grapevine DNA. The reference spectrum was subtracted from the SERS spectra of DNA samples, respectively, and baseline correction was applied, afterwards. The effects and importance of pre-processing Raman spectra in classifying PCA models are described elsewhere [48]. The most accurate grouping of the eight sets of spectral data was achieved when the original spectra were processed using multiplicative scattering correction (MSC) prior to PCA analysis.

**Figure 5:**
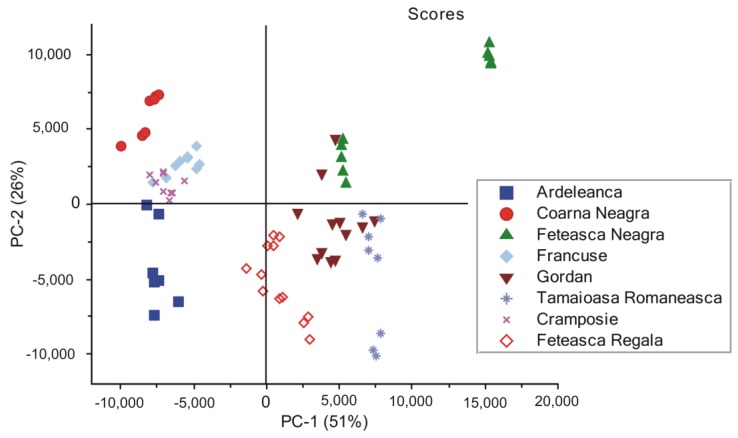
Principal component analysis (PCA) scores showing the grouping of the eight sets of SERS spectra of DNAs extracted from different grapevine varieties (Ardeleanca, Coarna Neagra, Feteasca Neagra, Francuse, Gordan, Tamaioasa Romaneasca, Cramposie, Feteasca Regala), respectively.

The PCA analysis was assessed by using 20 principal components (PCs), and the overall variance obtained was 98.89%. The first two PCs explained most of the variance in the sample grouping, and for this reason, they were selected for the PCA scores and loadings plots. The PCA score plot of the first two principal components shown in Figure 5 fairly discriminates between the eight groups of spectra, corresponding to the grapevine varieties. The score plot explains 77% of the spectral differences present in the 300–1800 cm^−1^ wavenumber region, considered as the spectral fingerprint of DNA.

Here, we point out that DNAs from Feteasca Neagra and Tamaioasa Romaneasca spectra in Figure 1, respectively, display very similar spectral features in the high wavenumber region of their profiles. However, they seem to be further discriminated, forming two distinct clusters each (Figure 5). An explanation for this might be the low wavenumber ranges of these SERS signals, presenting significant spectral differences, which could be responsible for this. The present observation underlines the efficiency of the discrimination method used by us.

**Figure 6:**
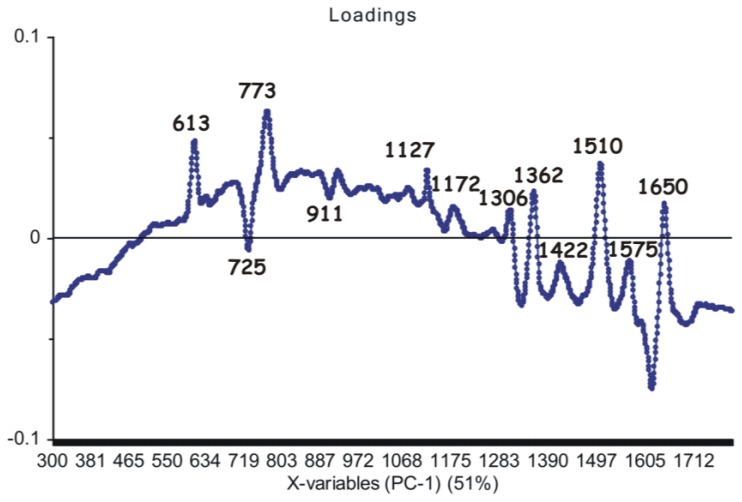
First principal component (PC1) loadings showing the marker bands considered as the main contribution (51% explained by the PCA model) in the grouping of the spectral data.

**Figure 7:**
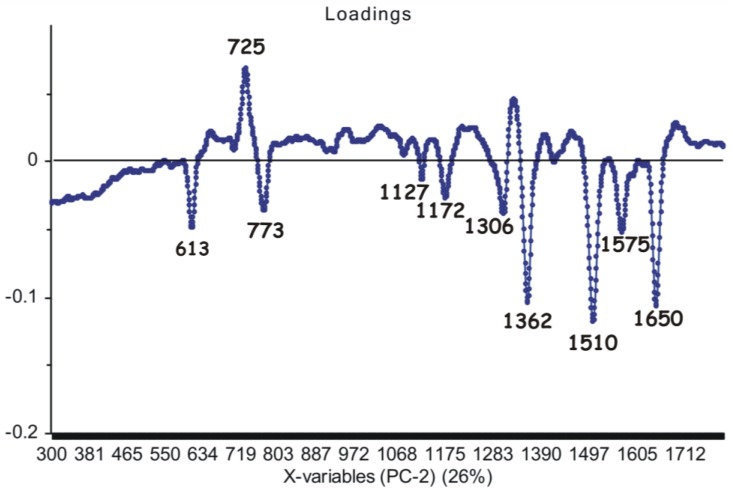
Second principal component (PC2) loadings showing the marker bands considered as the main contribution (26% explained by the PCA model) in the grouping of the spectral data.

Upon analyzing loadings charts (Figure 6 and Figure 7), it can be observed that the highest contribution in discrimination is correlated with the SERS marker bands, present with different relative intensities in all spectra: 613 cm^−1^, 725 cm^−1^, 773 cm^−1^, 911 cm^−1^, 1127 cm^−1^, 1172 cm^−1^, 1306 cm^−1^, 1362 cm^−1^, 1422 cm^−1^, 1510 cm^−1^, 1575 cm^−1^ and 1650 cm^−1^, respectively.

## 4. Conclusions

In this work, surface-enhanced Raman spectra of ten genomic DNAs extracted from leaf tissues of different grapevine (*Vitis vinifera* L.) varieties (Feteasca Neagra, Tamaioasa Romaneasca, Gordan, Feteasca Regala, Coarna Neagra, Ardeleanca, Braghina, Carloganca, Francuse and Cramposie), respectively, have been analyzed in the wavenumber range 300–1800 cm^−1^. Furthermore, structural modifications induced in plant DNAs upon femtosecond (170 fs) IR laser pulses irradiation (λ = 1100 nm) are discussed in detail for seven genomic DNAs extracted from Feteasca Regala, Braghina, Francuse, Carloganca, Feteasca Neagra, Tamaioasa Romaneasca and Ardeleanca, respectively.

The main marker bands were identified, and their corresponding assignments are summarized as follows: 452 cm^−1^ (stretching deoxyribose), 613 cm^−1^ (deoxyribose, C3′-*endo-*anti), 773 cm^−1^ (dC, C2′-*endo*-anti), 928 cm^−1^ (deoxyribose), 1127 cm^−1^ (dA (C8–N9 stretching, N9–H, C8–H deformation)), 1182 cm^−1^ (dA, dG (C5–C6 stretching)), 1311 cm^−1^ (dA, dG (C–N stretching)), 1363 cm^−1^ (dT, dA, dG (C–N stretching)), 1421 cm^−1^ (deoxyribose C2′H_2_ scissoring), 1510 cm^−1^ (dA (NH_2_ deformation)), 1575 cm^−1^ (dG, dA (C2=N3 of guanine)) and 1651 cm^−1^ (dT, δ(H_2_O), (C=O stretching; C=C stretching)).

The present SERS spectra, corresponding to genomic DNAs extracted from leaf tissues of different grapevine varieties, respectively, show precise profiles, providing useful information concerning molecular structure. Besides, we highlight the absence of some very intense SERS bands in the selected spectra of irradiated DNA samples (e.g., Feteasca Neagra, Tamaioasa Romaneasca, Ardeleanca). Loss of essential structural organization, including the alteration of DNA double helix, might be found in these cases. The observed grapevine variety-dependent sensitivity of nucleic acids to laser treatment might be correlated with the structural differences of genomic DNAs (e.g., sequence, base pairs content and number).

Feteasca Neagra grapevine variety DNA is the most structurally-responsive system to the femtosecond IR laser irradiation process.

Particularly, both C2′-*endo*-anti and C3′-*endo-*anti conformations have been detected in our nucleic acid systems. As an overall remark, the spectral range between 1500 and 1660 cm^−1^, being mostly due to dG, dA, dC and dT residues, seems to suffer significant changes upon the irradiation process. This finding could reflect changes in the base-stacking interactions of DNA.

Spectral shifts are mainly attributed to purines (dA, dG) and deoxyribose. Pyrimidine residues seem to be less affected by IR laser pulses irradiation. Furthermore, changes in the conformational properties of some nucleic acid segments are to be observed after femtosecond laser treatment.

In addition, using unbiased computational resources by means of principal component analysis (PCA), eight grapevine varieties were discriminated.

Further, cancer research could benefit from ultra-fast lasers technology by extending this study to tumor DNA. Besides, some research teams might be planning to study how intense the impact of femtosecond pulses is on cancerous cells, minimizing the risk of disease being caused.

## Figures and Tables

**Table 1:** Surface-enhanced Raman spectroscopy (SERS) wavenumbers (cm^−1^)* and tentative assignments of genomic DNAs extracted from different grapevine varieties (see text for details).

Feteasca Neagra	Tamaioasa Romaneasca	Gordan	Feteasca Regala	Coarna Neagra	Ardeleanca	Braghina	Carloganca	Francuse	Cramposie	Tentative Assignment ^a^
360 m	359 w	356 vw			363 w	354 w		362 m	359 vw	dG, dT, dA str
		378 w			421 vw			410 w		str dG, dT, dA
452 m	451 vw		448 w		449 w	456 w		448 m		str deoxyribose
						480 w	481 w			str deoxyribose
525 m	528 w		526 w		517 vw			530 w		dC/dT (ring def)
	571 w				570 w			577 w		dA (C2–H, N9–H wagging)
613 s	613 s	613 m	613 m		613 m					deoxyribose (C3′-*endo-*anti)
660 w				657 w	663 w					dG (ring breathing)
	707 vw					706 w			706 w	dA (ring breathing)
				732 s	725 m			721 w	723 vw	dA (ring breathing)
								741 vw		dT (ring breathing)
773 s	773 s	773 m	773 m		772 m	771 vw				dC, C2′-*endo*-anti
					795 m			796 m	790 w	bk 5′C–O–P–O–C3′ sym str
								829 w		deoxyribose C2′-*endo*
928 w	929 w		913 w		911 m			915 m	907 w	deoxyribose
				958 w						dA/dC/dG (NH_2_ rk)
1016 vw					988 vw			1008 w		dT (out-of-plane NH_2_ wagging)
				1029 vw	1055 vw			1031 w	1059 w	dA (N- deoxyribose str)
1086 w	1087 w	1085 vw			1088 vw	1084 vw	1084 w		1084 w	bk PO_2_^−^ sym str
1127 m	1127 m	1129 m	1122 w	1136 vw	1126 m	1127 m	1127 m	1119 vw	1127 m	dA (C8–N9 str, N9–H, C8–H def)
1182 s	1180 m	1175 m	1175 m		1176 s	1174 w	1170 m	1174 s	1172 m	dA, dG (C5–C6 str)
						1228 w	1230 w			dT (in-plane ring - CH_3_ str)
							1280 sh			dC (C–NH_2_ str + ring str)
1311 s	1310 s	1308 m	1310 m		1308 s	1306 m	1304 m	1300 m	1305 m	dA, dG (C–N str (Im))
				1331 m						dA, dG (ring mode)
1363 vs	1363 vs	1363 s	1363 vs	1383 w	1363 vs	1359 s	1361 m	1370 s	1362 m	dT, dA, dG (C–N str (Py))
1421 w	1422 m	1425 w	1423 vw					1432 w	1434 vw	Deoxyribose C2′H_2_ scissoring
		1468 vw	1451 vw	1465 w	1450 w		1452 w			Deoxyribose CH_2_
1510 vs	1510 vs	1510 vs	1509 vs		1510 vs	1508 m	1510 w		1512 w	dA (NH_2_ def)
				1545 vw	1534 m			1532 w		dT (in-plane ring str)
1575 s	1574 s	1575 m	1574 m	1575 vw	1576 m			1575 m		dG, dA (C2=N3 of guanine)
	1601 sh		1621 w		1621 s	1602 s	1604 s	1621 s	1623 m	dA, dG, dC (NH_2_ def)
1651 vs	1650 vs	1651 vs	1650 s		1650 s	1648 m				dT, δ(H_2_O) (C=O str, C=C str)
				1700 w						dG (C–O), C–C

* Abbreviations: vs, very strong; s, strong; m, moderate; w, weak; vw, very weak; sh, shoulder. ^a^ Abbreviations: dA, deoxyadenosine; dG, deoxyguanosine; dC, deoxycytidine; dT, thymidine; bk, backbone; sym, symmetric; str, stretching; def, deformation; rk, rocking; Im, imidazole; Py, pyrimidine.

**Table 2:** SERS wavenumbers (cm^−1^) * and tentative assignments of femtosecond infrared (IR) laser pulses-irradiated DNAs, extracted from different grapevine varieties (see text for details).

Feteasca Regala IR	Braghina IR	Francuse IR	Carloganca IR	Feteasca Neagra IR	Tamaioasa Romaneasca IR	Ardeleanca IR	Tentative Assignment ^a^
	354 w						dG, dT, dA str
453 w		454 m	462 m	441 w		459 m	str deoxyribose
473 w	482 w	480 m	482 m	481 w		480 m	str deoxyribose
	544 w						dC/dT (ring def)
558 w	569 w						dA (C2–H, N9–H wagging)
612 m	612 m		612 m	620 w			Deoxyribose (C3′-*endo-*anti)
661 w		641 w					dG (ring breathing)
723 w	707 w	706 w		706 w			dA (ring breathing)
		756 vw					dT (ring breathing)
772 m	773 m		775 m	778 w		769 w	dC, C2′-*endo*-anti
807 w		815 w	818 w			803 w	bk 5′C–O–P–O–C3′ sym str
			851 w				deoxyribose C3′-*endo*
911 m		915 w	924 w		915 w	911 m	Deoxyribose
	1083 m	1083 w	1086 w	1083 w	1084 w	1087 w	bk PO_2_^−^ sym str
1126 m	1127 m	1127 s	1126 m	1127 w		1123 w	dA (C8–N9 str, N9–H, C8–H def)
1177 s	1191 m	1172 m	1180 m	1172 w	1181 m	1171 m	dA, dG (C5–C6 str)
	1220 vw	1239 vw	1240 w				dT (ring str), dA, dC, dG (C8–N9 str)
	1278 w	1266 w					dC (C-NH_2_ str + ring str)
1310 m	1314 m		1314 m		1303 w	1304 m	dA, dG (C–N str (Im))
1363 vs	1364 vs	1363 m	1363 s	1361 w	1365 m	1365 m	dT, dA, dG (C–N str (Py))
	1425 w					1423 w	Deoxyribose C2′H_2_ scissoring
1442 w							Deoxyribose CH_2_
1510 s	1510 s		1512 s	1509 w	1509 w	1513 m	dA (NH_2_ def)
		1553 sh					dT (in-plane ring str)
1578 m	1574 m	1590 m	1578 m	1587 vw		1581 m	dG, dA (C2=N3 of guanine)
1623 s		1626 s			1625 s	1624 s	dA, dG, dC (NH_2_ def)
1650 s	1651 vs		1651 s	1653 w	1651 w	1650 s	dT, δ(H_2_O) (C=O str, C=C str)
	1683 vw						dA (NH_2_ scissoring)

* Abbreviations: vs, very strong; s, strong; m, moderate; w, weak; vw, very weak; sh, shoulder. ^a^ Abbreviations: dA, deoxyadenosine; dG, deoxyguanosine; dC, deoxycytidine; dT, thymidine; bk, backbone; sym, symmetric; str, stretching; def, deformation; Im, imidazole; Py, pyrimidine.

**Table 3:** Main wavenumber shifts and tentative assignments of SERS marker bands, characterizing femtosecond infrared (IR) laser pulses-irradiated (1100 nm) genomic DNAs from different grapevine varieties, as compared to the corresponding marker modes of untreated samples, respectively.

Grapevine Varieties DNA (cm^−1^)	Shift (cm^−1^)	Grapevine varieties IR Irradiated DNA (cm^−1^)	Tentative Assignments ^a^
Feteasca Regala		Feteasca Regala IR	
1423	+19	1442	Deoxyribose C2′H_2_ scissoring
Braghina		Braghina IR	
1174	+17	1191	dA, dG (C5–C6 str)
1228	−8	1220	dT (in-plane ring –CH_3_ str)
1306	+8	1314	dA, dG (C–N str (Im))
1602	−28	1574	dA, dG, dC (NH_2_ def)
Francuse		Francuse IR	
721	−15	706	dA (ring breathing)
741	+15	756	dT (ring breathing)
796	+19	815	bk 5′C–O–P–O–C3′ sym str
1119	+8	1127	dA (C8–N9 str, N9–H, C8–H def)
1532	+21	1553	dT (in-plane ring str)
1575	+15	1590	dG, dA (C2=N3 of guanine)
Carloganca		Carloganca IR	
1170	+10	1180	dA, dG (C5–C6 str)
1230	+10	1240	dT (in-plane ring –CH_3_ str)
1304	+10	1314	dA, dG (C–N str (Im))
1604	−26	1578	dA, dG, dC (NH_2_ def)
Feteasca Neagra		Feteasca Neagra IR	
452	−11	441	str deoxyribose
1182	−10	1172	dA, dG (C5–C6 str)
1575	+12	1587	dG, dA (C2=N3 of guanine)
Tamaioasa Romaneasca		Tamaioasa Romaneasca IR	
929	−14	915	Deoxyribose
1601	+24	1625	dA, dG, dC (NH_2_ def)
Ardeleanca		Ardeleanca IR	
449	+10	459	str deoxyribose
795	+8	803	bk 5′C–O–P–O–C3′ sym str
1450	−27	1423	Deoxyribose CH_2_

^a^ Abbreviations: dA, deoxyadenosine; dG, deoxyguanosine; dC, deoxycytidine; dT, thymidine; bk, backbone; sym, symmetric; str, stretching; def, deformation; Im, imidazole.

## Supplementary Materials

The following are available online at http://www.mdpi.com/2079-4991/6/6/96/s1.

## References

[1] Tomas M., Blumhardt P., Deutzmann A., Schwarz T., Kromm D., Leitenstorfer A., Ferrando-May E. (2013). Imaging of the DNA damage-induced dynamics of nuclear proteins via nonlinear photoperturbation. J. Biophoton..

[2] Cho S.-H., Chang W.-S., Kim J.-G., Whang K.-H., Choi K.-S., Sohn S.-H. (2008). *In situ* observation of photo-bleaching in human single living cell excited by a NIR femtosecond laser. Appl. Surf. Sci..

[3] Russmann C., Stollhof J., Weiss C., Beigang R., Beato M. (1998). Two wavelength femtosecond laser induced DNA-protein crosslinking. Nucleic Acids Res..

[4] Mahajan S., Richardson J., Brown T., Bartlett P.N. (2008). SERS-melting: A new method for discriminating mutations in DNA sequences. J. Am. Chem. Soc..

[5] Fabris L., Dante M., Braun G., Lee S.J., Reich N.O., Moskovits M., Nguyen T.-Q., Bazan G.C. (2007). A heterogeneous PNA-based SERS method for DNA detection. J. Am. Chem. Soc..

[6] He Y., Su S., Xu T., Zhong Y., Zapien J.A., Li J., Fan C., Lee S.-T. (2011). Silicon nanowires-based highly-efficient SERS-active platform for ultrasensitive DNA detection. Nano Today.

[7] Panikkanvalappil S.R., Mackey M.A., El-Sayed M.A. (2013). Probing the unique dehydration-induced structural modifications in cancer cell DNA using surface-enhanced Raman spectroscopy. J. Am. Chem. Soc..

[8] Muntean C.M., Leopold N., Tripon C., Coste A., Halmagyi A. (2015). Surface-enhanced Raman spectroscopy of genomic DNA from *in vitro* grown tomato (*Lycopersicon esculentum* Mill.) cultivars before and after plant cryopreservation. Spectrochim. Acta A.

[9] Qiu L., Liu P., Zhao L., Wen M., Yang H., Fan S., Zhou L. (2014). Analysis of plant genomic DNAs and the genetic relationship among plants by using surface-enhanced Raman spectroscopy. Vib. Spectrosc..

[10] Muntean C.M., Leopold N., Halmagyi A., Valimareanu S. (2011). Surface-enhanced Raman spectroscopy of genomic DNA from *in vitro* grown plant species. J. Raman Spectrosc..

[11] Gout E., Bligny R., Douce R. (1992). Regulation of intracellular pH values in higher-plant cells. Carbon-13 and phosphorus-31 nuclear-magnetic-resonance studies. J. Biol. Chem..

[12] Muntean C.M., Leopold N., Halmagyi A., Valimareanu S. (2011). Surface-enhanced Raman spectroscopy of DNA from leaves of *in vitro* grown apple plants. J. Raman Spectrosc..

[13] Muntean C.M., Leopold N., Halmagyi A., Valimareanu S. (2013). Surface-enhanced Raman scattering assessment of DNA from leaf tissues adsorbed on silver colloidal nanoparticles. J. Raman Spectrosc..

[14] Muntean C.M., Halmagyi A., Puia M.D., Pavel I. (2009). FT-Raman signatures of genomic DNA from plant tissues. Spectrosc-Int. J..

[15] Sun L., Song Y., Wang L., Guo C., Sun Y., Liu Z., Li Z. (2008). Ethanol-induced formation of silver nanoparticle aggregates for highly active SERS substrates and application in DNA detection. J. Phys. Chem. C.

[16] Deng H., Bloomfield V.A., Benevides J.M., Thomas G.J. (1999). Dependence of the Raman signature of genomic B-DNA on nucleotide base sequence. Biopolymers.

[17] Wu C.-Y., Lo W.-Y., Chiu C.-R., Yang T.-S. (2006). Surface-enhanced Raman spectra of oligonucleotides induced by spermine. J. Raman Spectrosc..

[18] Ke W., Zhou D., Wu J., Ji K. (2005). Surface-enhanced Raman spectra of calf thymus DNA adsorbed on concentrated silver colloid. Appl. Spectrosc..

[19] Sun L., Sun Y., Xu F., Zhang Y., Yang T., Guo C., Liu Z., Li Z. (2009). Atomic force microscopy and surface-enhanced Raman scattering detection of DNA based on DNA–nanoparticle complexes. Nanotechnology.

[20] Pagba C.V., Lane S.M., Wachsmann-Hogiu S. (2010). Raman and surface-enhanced Raman spectroscopic studies of the 15-mer DNA thrombin-binding aptamer. J. Raman Spectrosc..

[21] Treffer R., Lin X., Bailo E., Deckert-Gaudig T., Deckert V. (2011). Distinction of Nucleobases—A Tip-Enhanced Raman Approach. Beilstein J. Nanotechnol..

[22] Wartell R.M., Harrell J.T. (1986). Characteristics and variations of B-type DNA conformations in solutions: A quantitative analysis of Raman band intensities of eight DNAs. Biochemistry-US.

[23] Ruiz-Chica A.J., Medina M.A., Sánchez-Jiménez F., Ramírez F.J. (2004). On the interpretation of Raman spectra of 1-aminooxy-spermine/DNA complexes. Nucleic Acids Res..

[24] Muntean C.M., Nalpantidis K., Feldmann I., Deckert V. (2009). Zn^2+^-DNA interactions in aqueous systems: A Raman spectroscopic study. Spectrosc-Int. J..

[25] Rajani C., Kincaid J.R., Petering D.H. (1999). The presence of two modes of binding to calf thymus DNA by metal-free bleomycin: A low frequency Raman study. Biopolymers.

[26] Muntean C.M., Misselwitz R., Dostál L., Welfle H. (2006). Mn^2+^–DNA interactions in aqueous systems: A Raman spectroscopic study. Spectroscopy.

[27] Dostál L., Misselwitz R., Welfle H. (2005). Arc repressor−operator DNA interactions and contribution of Phe10 to binding specificity. Biochemistry-US.

[28] Thomas G.J., Benevides J.M., Duguid J., Bloomfield V.A., Theophanides T., Anastassopoulou J., Fotopoulos N. (1993). Roles of cations in the structure, stability and condensation of DNA. Fifth International Conference on the Spectroscopy of Biological Molecules.

[29] Puppels G.J., Otto C., Greve J., Robert-Nicoud M., Arndt-Jovin D.J., Jovin T.M. (1994). Raman microspectroscopic study of low-pH-induced changes in DNA structure of polytene chromosomes. Biochemistry-US.

[30] Muntean C.M., Puppels G.J., Greve J., Segers-Nolten G.M.J. (2002). Influence of Ca^2+^ cations on low pH-induced DNA structural transitions. Biopolymers.

[31] Thomas G.J., Benevides J.M., Overman S.A., Ueda T., Ushizawa K., Saitoh M., Tsuboi M. (1995). Polarized Raman spectra of oriented fibers of A DNA and B DNA: Anisotropic and isotropic local Raman tensors of base and backbone vibrations. Biophys. J..

[32] Muntean C.M., Puppels G.J., Greve J., Segers-Nolten G.M.J., Cinta-Pinzaru S. (2002). Raman microspectroscopic study on low-pH-induced DNA structural transitions in the presence of magnesium ions. J. Raman Spectrosc..

[33] Braun C.S., Jas G.S., Choosakoonkriang S., Koe G.S., Smith J.G., Middaugh C.R. (2003). The structure of DNA within cationic lipid/DNA complexes. Biophys. J..

[34] Guan Y., Wurrey C.J., Thomas G.J. (1994). Vibrational analysis of nucleic acids. I. The phosphodiester group in dimethyl phosphate model compounds: (CH_3_O)_2_PO^2−^, (CD_3_O)_2_PO^2−^, and (^13^CH_3_O)_2_PO^2^. Biophys. J..

[35] Duguid J., Bloomfield V.A., Benevides J., Thomas G.J. (1993). Raman spectroscopy of DNA-metal complexes. I. Interactions and conformational effects of the divalent cations: Mg, Ca, Sr, Ba, Mn, Co, Ni, Cu, Pd, and Cd. Biophys. J..

[36] Muntean C.M., Dostál L., Misselwitz R., Welfle H. (2005). DNA structure at low pH values, in the presence of Mn^2+^ ions: A Raman study. J. Raman Spectrosc..

[37] Tsuboi M., Benevides J.M., Thomas G.J. (2007). The complex of ethidium bromide with genomic DNA: Structure analysis by polarized Raman spectroscopy. Biophys. J..

[38] Neault J.F., Naoui M., Manfait M., Tajmir-Riahi H.A. (1996). Aspirin-DNA interaction studied by FTIR and laser Raman difference spectroscopy. FEBS Lett..

[39] De Gelder J., De Gussem K., Vandenabeele P., Moens L. (2007). Reference database of Raman spectra of biological molecules. J. Raman Spectrosc..

[40] Huser T., Orme C.A., Hollars C.W., Corzett M.H., Balhorn R. (2009). Raman spectroscopy of DNA packaging in individual human sperm cells distinguishes normal from abnormal cells. J. Biophoton..

[41] Xie W., Ye Y., Shen A., Zhou L., Lou Z., Wang X., Hu J. (2008). Evaluation of DNA-targeted anti-cancer drugs by Raman spectroscopy. Vib. Spectrosc..

[42] Willets K. (2009). Surface-enhanced Raman scattering (SERS) for probing internal cellular structure and dynamics. Anal. Bioanal. Chem..

[43] Jaunich M., Raje S., Kim K., Mitra K., Guo Z. (2008). Bio-heat transfer analysis during short pulse laser irradiation of tissues. Int. J. Heat Mass Transfer.

[44] Mao H., Arias-Gonzalez J.R., Smith S.B., Tinoco I., Bustamante C. (2005). Temperature control methods in a laser tweezers system. Biophys. J..

[45] Hung M.-S., Kurosawa O., Washizu M. (2012). Single DNA molecule denaturation using laser-induced heating. Mol. Cell. Probes.

[46] Miljanić S., Dijanošić A., Matić I. (2015). Adsorption mechanisms of RNA mononucleotides on silver nanoparticles. Spectrochim. Acta A.

[47] Mircescu N., Zhou H., Leopold N., Chiş V., Ivleva N., Niessner R., Wieser A., Haisch C. (2014). Towards a receptor-free immobilization and SERS detection of urinary tract infections causative pathogens. Anal. Bioanal. Chem..

[48] Heraud P., Wood B.R., Beardall J., McNaughton D. (2006). Effects of pre-processing of Raman spectra on *in vivo* classification of nutrient status of microalgal cells. J. Chemometr..

