# Supplementary material for: Structural Changes Induced in Grapevine (Vitis vinifera L.) DNA by Femtosecond IR Laser Pulses: A Surface-Enhanced Raman Spectroscopic Study
Source: Nanomaterials (Basel). 2016 May 25;6(6):96. doi: 10.3390/nano6060096 (PMC5302626; doi:10.3390/nano6060096)
Supplement: Supplementary file 1 [file nanomaterials-06-00096-s001.pdf]

Supplementary Materials: Structural Changes Induced in Grapevine (*Vitis vinifera* L.) DNA by Femtosecond IR Laser Pulses: A Surface-Enhanced Raman Spectroscopic Study

Nicoleta E. Dina, Cristina M. Muntean, Nicolae Leopold, Alexandra Fălămaş, Adela Halmagyi and Ana Coste

Table S1. Grapevine (*Vitis vinifera* L.) DNA amount (non-irradiated samples) in the colloidal suspensions for surface-enhanced Raman spectroscopic measurements. No.: number.

No.	Grapevine Varieties	Genomic DNA Amount ng/μL
1.	Ardeleanca	0.72
2.	Braghina	2.97
3.	Carloganca	2.16
4.	Coarna Neagra	14.12
5.	Cramposie	2.37
6.	Feteasca Neagra	1.13
7.	Feteasca Regala	2.70
8.	Francuse	1.52
9.	Gordan	0.94
10.	Tamaioasa Romaneasca	1.64

Table S2. Grapevine (*Vitis vinifera* L.) DNA amount (irradiated samples) in the colloidal suspensions for surface-enhanced Raman spectroscopy (SERS) measurements.

No.	Grapevine Varieties	Genomic DNA Amount ng/μL
1.	Ardeleanca IR	0.78
2.	Braghina IR	0.44
3.	Carloganca IR	0.92
4.	Feteasca Neagra IR	1.94
5.	Feteasca Regala IR	1.10
6.	Francuse IR	1.46
7.	Tamaioasa Romaneasca IR	0.46

UV-Vis characterization of DNA-silver nanoparticles complexes

Ultraviolet-visible spectrophotometry (UV-Vis) was used to characterize the produced silver colloid and DNA-Ag nanoparticles systems (Figures S1 and S2). The wavelength position of the extinction maximum of the recorded UV-Vis spectrum of silver colloidal system, was found at 405 nm, providing information on the average particle size of approximately 25 nm. Besides, its full width at half-maximum (FWHM) (around 100 nm) can be used to estimate particle dispersion, which in this case is rather monodispersed [1,2].

UV-Vis absorption spectra of Ag colloid and DNA-silver nanoparticles systems were recorded with a Jasco V630 spectrophotometer (JASCO International Co., Ltd., Hachioji, Tokyo, Japan). Extinction spectra of silver colloid–DNA systems (non-irradiated and irradiated samples), respectively, for different grapevine varieties, are presented in Figures S1 and S2. λ_{max} (nm) is indicated for each UV-Vis absorption spectrum.

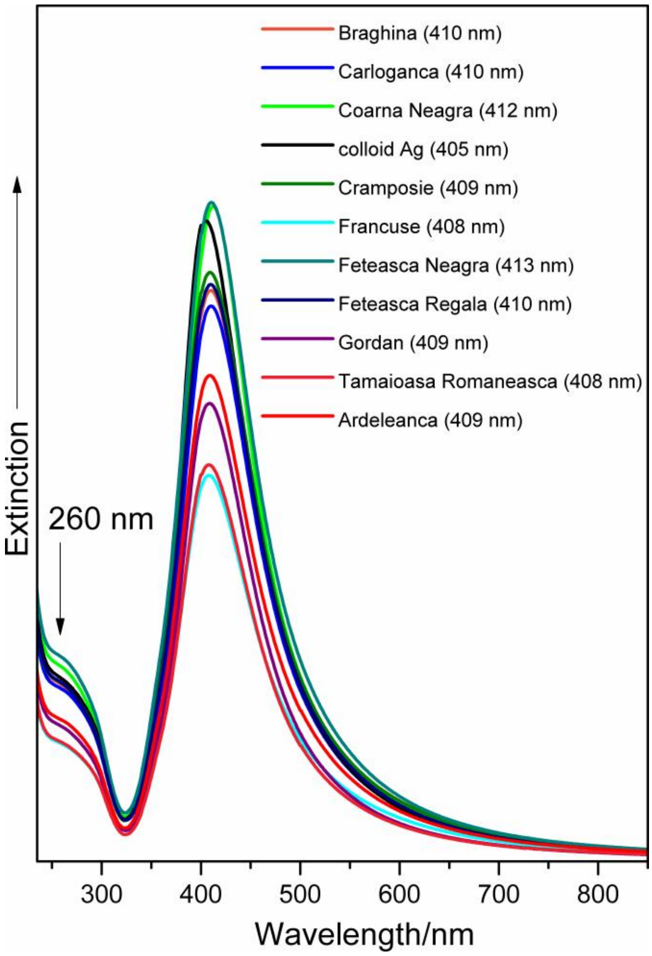

Figure S1. Extinction spectra of silver colloid-DNA systems (non-irradiated nucleic acids samples) for different grapevine varieties, respectively. λ_{max} (nm) is indicated in each case. Ultraviolet-visible spectrophotometry (UV-Vis) absorbance of Ag colloid is presented for comparison.

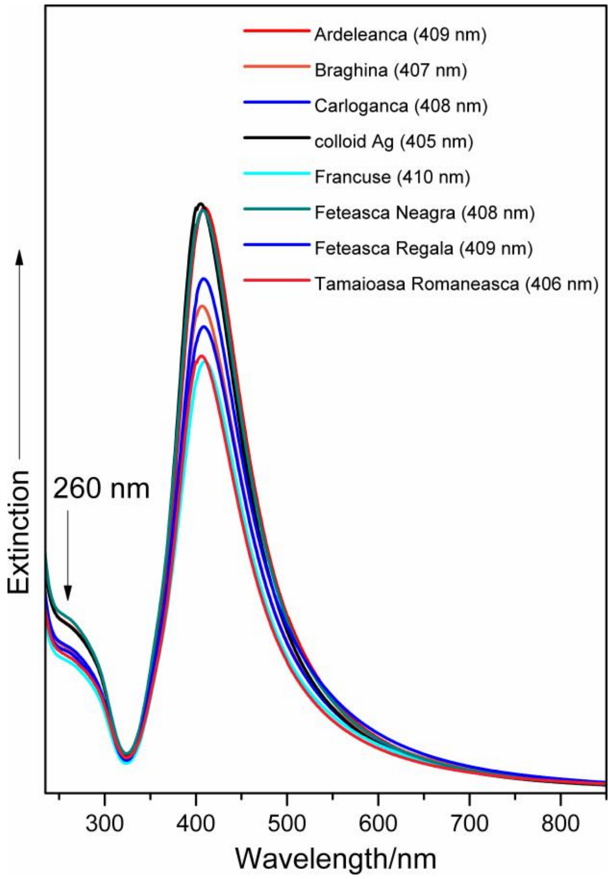

Figure S2. Extinction spectra of silver colloid-DNA systems (irradiated nucleic acids samples) for different grapevine varieties, respectively. λ_{max} (nm) is indicated in each case. UV-Vis absorbance of Ag colloid is presented for comparison.

It has been observed, that in most cases, upon DNAs laser treatment, λ_{max} (nm) is shifted to lower wavelengths. These shifts are not quite significant. However we can suppose that some structural changes in DNA, mostly due to modified purinic (dG, dA) residues and some molecular geometry changes of nucleic acids, due to the interaction with the Ag surface, might be responsible for these shifts.

References

1. Shelton, R.D.; Haas, J.W.; Wachter, E.A. Surface-enhanced Raman detection of aqueous cyanide. *Appl. Spectrosc.* **1994**, *48*, 1007–1010.
2. Leopold, N. *Surface-Enhanced Raman Spectroscopy*; Napoca Star: Cluj-Napoca, Romania, 2009.

© 2016 by the authors; licensee MDPI, Basel, Switzerland. This article is an open access article distributed under the terms and conditions of the Creative Commons by Attribution (CC-BY) license (http://creativecommons.org/licenses/by/4.0/).
